# Perspectives on decisions for treatment and care in severe asthma

**DOI:** 10.1016/j.waojou.2020.100500

**Published:** 2021-01-16

**Authors:** Tonya Winders, Jorge Maspero, Luke Callan, Mona Al-Ahmad

**Affiliations:** a Allergy & Asthma Network/Global Allergy & Airways Patient Platform (GAAPP), Vienna, VA, USA; b Allergy and Respiratory Research Unit, Fundación CIDEA, Buenos Aires, Argentina; c Global Market Access and Pricing, AstraZeneca UK Ltd., Cambridge, UK; d Department of Microbiology, Faculty of Medicine, Kuwait University, Kuwait

**Keywords:** Burden, Perspective, Severe asthma

## Abstract

**Background:**

Severe asthma is a subtype of asthma that can be hard to control, resulting in an exceptional impact on an individual's quality of life. The aim of this review article is to explore the misalignment of perceptions of severe asthma among different stakeholders to identify how to reduce burden and improve delivery of care.

**Results:**

The misalignment of perspectives is best reflected in randomised controlled trials (RCTs) in asthma treatments, which are often designed for regulatory approval with a focus on exacerbations with no direct input from the individuals that the treatments are designed for. Based on a literature review and the clinical experience of the authors to overcome this disparity, the goals of people with severe asthma need to be incorporated throughout their care, from study design to the day-to-day management of their condition. Improved education for individuals and their support network will provide them with resources and knowledge so that they can effectively communicate their needs to other stakeholders involved in their care.

**Conclusion/recommendation:**

A collaborative effort from all stakeholders is essential to ensure efficient management of asthma and a reduction in asthma burden on individuals and society.

## Background

Despite the increasing prevalence of severe asthma worldwide, the condition remains uncontrolled in many individuals.^1^^,^^2^ The day-to-day struggles suffered by people with asthma affect not only the individual, but also their caregivers, and they are associated with increased direct (eg, hospital admissions) and indirect (eg, lost income owing to absence from work) health care costs to the local health care system and wider society.^1^^,^^3^, ^4^, ^5^, ^6^ As the main aim of health care services is to improve the health and wellbeing of the population they serve, it is vital that people with asthma are involved through shared decision-making around their treatment and care (unpublished observation; data on file).^7^ However, this is not always the case, which creates an unsatisfactory patient experience, resulting in poor treatment outcomes.^8^

There is a plethora of guidelines available on the recommendations for the treatment and care of severe asthma globally, such as those from the Global Initiative for Asthma (GINA)^2^ and the international European Respiratory Society/American Thoracic Society.^9^ Despite their wide availability, there are challenges to making the information available to all asthma stakeholders and implementing these recommendations at a local level.^2^^,^^10^ We have defined stakeholders as any group, organisation, company, or individual with an interest (financial or personal) in asthma, which includes people with asthma, their caregivers, policymakers, payer organisations, and patient advocacy groups.^11^ Severe asthma management should be a multidisciplinary effort among all stakeholders, taking into account national and local barriers, such as financial constraints and communication issues.^12^

To improve care, people with severe asthma would benefit from collaborative relationships with other partners who can help focus on patient-centred endpoints in clinical trials for asthma therapies (unpublished observation; data on file).^7^^,^^13^ The inclusion of the perspective of people with severe asthma can promote engagement, trust, and understanding to aid communication to the individual on their needs and education on complex medical terms.^14^ It is imperative to include the views of individuals at the earliest opportunity to allow the assessment of new care strategies for addressing existing unmet needs in the delivery of severe asthma care.

Previously, 3 focus groups were run with people in severe asthma in the United Kingdom, Germany and the United States to develop the initial hypothesis on the misalignment of perspectives among severe asthma stakeholders (unpublished observation; data on file).^7^ The perspectives of people with severe asthma regarding their treatment and care are not being taken into account consistently (unpublished observation; data on file).^7^ This unmet need must be addressed urgently.^15^ This review, based on author experiences and current literature, aims to explore the extent of the misalignment of perspectives among stakeholders of severe asthma and identify whose responsibility it is to increase communication and involvement to improve outcomes that will not only benefit the individual but also the health care system.

## Methods

Based on previous research, a mixed-methods approach was used to explore engagement with people with severe asthma from all aspects of the delivery of severe asthma care.

### Literature review

A brief literature search was conducted using PubMed for English language articles published between November 2009 and November 2019 where the full text was available. Articles were identified using the following search terms: (asthma) AND (burden) AND (perception OR view OR perspective OR insight OR attitude). Articles were excluded if they focussed solely on paediatric asthma or related to irrelevant comorbidities (eg, fungal infection). Titles relating to asthma views, clinical trial design, and communication were included based on the following categories: the misalignment of perspectives in severe asthma, the lack of inclusion of patient views in randomised controlled trials (RCTs), inadequate prioritisation of patient needs, and the requirement for the improvement of stakeholder communication with patients. A total of 216 clinical papers were identified, and after screening for the relevant abstracts, 11 were included in the manuscript (see Supplemental Appendix 1).

### Stakeholder input

Additionally, relevant materials and articles were included from various sources known to the authors and from their experience as different severe asthma stakeholders. The views of all severe asthma stakeholder groups were considered, where possible, and the following were included in this review article: payers, clinicians, patient advocates, caregivers, and people with severe asthma. Recommendations for individual stakeholder groups were given where an unmet need was identified.

A data collection form was utilised to record key points from the articles sourced by authors and from the literature search (see Supplemental Appendix 1).

The limitations to this mixed-method approach include the possibility of missing relevant publications, as the literature search was not systematic; and while the views of the authors represent different stakeholder groups, they may not be representative of all views on asthma management and care.

## Results

This present review identified 4 key areas where there is a misalignment among stakeholders in severe asthma.

### Misalignment of perspectives in severe asthma

The burden of severe asthma is viewed differently between those with the disease and those involved in the delivery of their care (unpublished observation; data on file).^7^ As seen across many therapy areas including asthma, only the views of payers, providers, and policymakers are considered in treatment and service design, leaving the views of people with asthma and caregivers inadequately addressed (unpublished observation; data on file).^7^ However, these are important as they provide insight about the treatment and the day-to-day burden of the condition.^16^

The Observations of Patient Experience in the Nation (OPEN) asthma survey, conducted in the United States, demonstrated a significant disconnect in the belief of asthma control between people with asthma (n = 2900) and health care providers (n = 850).^17^ The majority of the health care providers (84%) believed that individuals with well-controlled asthma experienced no limitation in everyday activities, whereas 70% of those with well-controlled asthma reported that their disease affected certain aspects of their daily living, for example household chores and sleep.^18^ However, the misalignment of perspectives on asthma control is not only a result of physician underestimation of asthma severity; worldwide, people with asthma lack understanding on how well their condition is controlled and do not recognise that change in symptoms is an indicator of poor control, emphasising a disconnect between the guidelines and individuals’ perceptions of their own asthma.^8^

As the level of asthma control correlates with quality of life (QoL),^4^ communication has to improve between people with asthma and their health care providers, even at routine visits, to ensure appropriate treatment plans are created.^8^^,^^17^ Strategies to assess and improve patient-physician communications have been explored to reduce the asthma burden for individuals;^5^^,^^8^^,^^19^ in Latin America, technologies such as email have been suggested as a method of improving the frequency and speed of communication between the physician and patient.^19^ However, to ensure improvement in communication globally, it is essential that primary care physicians (PCPs) have training to ensure that they refer individuals with severe asthma to pulmonologists in secondary care, for example,^8^ and that they communicate with patients effectively to ensure understanding of their treatment pathway.^13^^,^^20^

The perspective of the caregiver is important but often overlooked. As severe asthma is a chronic disease, the impact on caregivers is considerable. The views of caregivers and others in the support network (for instance those who provide childcare during exacerbations or those who collect medication when the individual with asthma cannot do so) would help shape the delivery of health care services through initiatives such as multidisciplinary teams.^8^^,^^21^ It is also imperative for people with asthma and their support network to communicate effectively with each other, as criticism has the potential to negatively impact on an individual's asthma.^21^

Decisions on asthma treatment vary even among health care professionals.^22^ An online survey conducted in Mexico of physicians from different asthma-related specialities (283 allergists, 106 pulmonologists, 18 ear-nose-throat specialists, 161 paediatricians, and 44 general practitioners) found inconsistencies between the views on diagnoses and treatments across the different specialities.^22^ For example, when asked what therapeutic combination should be used for maintenance and rescue treatment in a fictitious mild but persistent case of asthma, many responses did not align with guideline advice.^22^ Physician-group differences were also evident in the answers; 15% of pulmonologists compared with ~45% of general practitioners (p < 0.001) erroneously suggested a treatment that was not aligned to guideline advice.^22^ People with severe asthma and their health care professionals should have access to best practice guidance on treatment and management of the condition, as suggested in the Patient Charter for Severe Asthma that was written by 12 asthma experts to improve care in severe asthma worldwide.^15^

It is critical not only to have access to evidence-based guidance but also to have the resource and collaborative input from people with severe asthma, as well as their caregivers and relevant stakeholders, to achieve improved asthma outcomes.^8^ Currently, the perspectives of these stakeholders are not included in global guidance and reports. The development of tools to help communicate individuals’ experiences and preferences to other stakeholders, both qualitatively and quantitatively, are required (unpublished observation; data on file)^7^ to shape care and guidance globally.^12^

Stakeholders have to keep up to date with relevant research at a global level not only for the benefit of individuals with severe asthma but also to understand global differences among clinical practices. The Patient Charter urges policymakers and advocates for better care to build consensus on what the treatment of severe asthma should look like in their local health care system and states that national guidelines should reflect changes to updated treatment options.^15^

### Lack of inclusion of patient views in randomised controlled trials

The inclusion of perspectives of patients in RCTs is critical to ensure the outcomes meet the needs of the intervention's target population. Most clinical studies include regulatory or research-orientated outcomes, rather than examining interventions from the patient and caregiver perspective.^13^ This perspective is important to the outcomes of RCTs and makes the patient experience meaningful in the long term.^13^ Individuals will then be considered not only as “subjects” in a trial but also as engaged stakeholders in their own treatment and care. Along with clinical effectiveness and safety, this experience is seen as a central outcome in certain regions (eg, the UK National Health Service), but this paradigm needs to be accepted worldwide.^13^

There is a difference in expectations between individuals with severe asthma and payers, regulators, and clinicians on asthma and what a new medicine should deliver (unpublished observation; data on file).^7^ Current RCTs involving treatments for severe asthma do not always place a focus on patient-centric endpoints or incorporate patients’ values into the design (unpublished observation; data on file),^7^ although it has been shown that patient-reported outcomes (as measured in the Asthma Quality of Life Questionnaire [AQLQ], for example) are aligned with clinical outcomes such as forced expiratory volume in 1 second (FEV_1_).^23^ And for regulatory approval, it is assumed that people with asthma would like new treatments to deliver only a reduction in exacerbations.^24^ Consequently, the evidence base does not show the extent of the impact on patient outcomes, as trials are not designed to capture this information as a primary endpoint, which is seen in systematic reviews of biologics.^24^ In order to accommodate the different perspectives, RCT design needs to take into account the views of multiple stakeholders, including people with severe asthma. In addition, the different endotypes of asthma are not considered in clinical trial design often enough.^25^ Inclusion and exclusion criteria are restrictive; consequently, many individuals with severe asthma cannot be included.^25^ Analyses exploring the external validity of RCTs have found that only ~10% of patients with severe asthma would be eligible for such trials.^26^^,^^27^ Other factors may also affect patient representation in trials, namely patient willingness or refusal to participate.^28^ To accommodate different perspectives, clinical trials should consider patient-relevant endpoints and be more representative of people with asthma seen in real-world clinical practice.

As severe asthma is a broad condition with many clinical phenotypes,^2^ it is hard to design a treatment goal that encompasses them all.^29^ However, as asthma remains uncontrolled despite several treatment options,^30^ it is certain that the current participants in clinical trials are not representative of those seen in clinical practice and are focused towards other stakeholder goals.^29^^,^^31^ That is why “real-life experience” and “real patient” representation have been powerful elements of recent data, and the validity of RCTs should be assessed to inform guideline development.^32^ A lack of representative participants impacts guidelines, which base their recommendations on clinical evidence and application to clinical practice.^31^ Steps must be taken to broaden the inclusion criteria of RCTs to represent wider asthma populations.^31^

An intuitive way to design RCTs with patient-relevant endpoints is to include those perspectives from the outset, from study design to outcome measures, which helps to identify whether a treatment is effectively meeting the needs of the individual.^33^ Although RCTs have recently included more patient-focused aspects and greater numbers of patient-reported outcomes (for example QoL) than ever before, there is still a lack of patient perspectives in newer therapies (eg, biologics) to determine not only how effective the drug is, but also how the therapy will impact daily life. For instance, anxiety and depression are frequently found in adults with severe asthma, and this negatively impacts on QoL.^34^ The prevalence of anxiety, depression, and panic disorders is higher in people with asthma than in matched controls and is associated with poor outcomes and death, but this is rarely measured in clinical trials for severe asthma treatments.^34^^,^^35^ Newer severe asthma biologic therapies have included various questionnaires on factors such as QoL in study designs;^36^, ^37^, ^38^ however, there is a need for patient-centred endpoints to be standardised across all asthma clinical trials.^39^

We should not, however, forget the impact of the placebo effect in severe asthma RCTs, including that seen on patient-reported outcomes, and the benefit patients receive from the administration of a structured asthma management regimen.^40^ It is interesting to note that this effect may not be so significant in oral corticosteroid (OCS)-sparing trials, since current tools overestimate the QoL in patients who are exposed to OCS.^41^

Patient insight should be encouraged, from early phase^42^ to phase 4 studies, and in the licensing of treatments and appraisal by Health Technology Assessment (HTA) bodies, in order to improve patient outcomes and therefore improve health care processes.^43^^,^^44^ In the United Kingdom, people with dermatology conditions are routinely involved from study design to dissemination of findings.^45^ A patient panel (based in the Centre of Evidence Based Dermatology at the University of Nottingham, UK) takes part in focus groups and also joins steering committees to ensure the views of individuals are considered throughout.^45^ Pharmaceutical companies have a responsibility and duty to improve the interface between themselves and individuals with asthma to enhance study outcomes and ultimately improve care and its delivery.

### Inadequate prioritisation of patient needs

Exacerbations are the predominant focus in decision-making in the severe asthma setting. Although the reduction of exacerbations is important to individuals and other stakeholders, such as hospitals, additional factors prioritised by people with asthma, such as QoL and symptoms, are overlooked or not incorporated into decision-making (unpublished observation; data on file).^7^ Across many therapies, a patient-focused approach is becoming increasingly popular in the economic evaluation of new treatments (unpublished observation; data on file). However, while a systematic literature review of economic evaluations has found an increase in studies involving this viewpoint in recent years, there is still an absence of views from people with severe asthma in the literature (unpublished observation; data on file). There must be an increase in patient-centric approaches not only in RCTs but also other areas, for example, health care policies and treatment regimens. For instance, one of the principles of the Severe Asthma Patient Charter is that individuals deserve a timely diagnosis by a multidisciplinary team that has access to all appropriate resources.^15^ This is a patient-centric approach that is essential for the improvement of QoL and reduction of asthma burden through a multifactorial approach of treating severe asthma.

The Patient Charter states that shortening the patient journey (from initial symptoms to accepting the disease) is important to improve QoL and avoids compromising on daily tasks to accommodate the individual's asthma.^15^ To facilitate this, individuals need to be aware of the treatment guidelines (for example, those of GINA) and other national and local recommendations.^9^^,^^30^ For instance, many people with severe asthma continue to use OCS as a long-term treatment for severe asthma, despite the undesirable physical and emotional side effects such as weight gain, fatigue, anxiety, diabetes, and osteoporosis.^21^^,^^46^ Similar results are also found in those who overuse short-acting β_2_-agonists (SABAs).^47^ If people overestimate how well their asthma is controlled, it can lead to excessive SABA use.^47^ It is necessary that people with severe asthma are advised on the misuse and benefit-risk profile of treatments such as this so that they can make informed and educated decisions in partnership with their health care professionals.^48^ Indeed, there are current efforts at minimising the overuse of OCS,^49^ also being included in the Patient Charter (Principle 5 “I deserve not to be reliant on OCS”).^15^ Awareness of patients' views on their treatments is important; understanding patient perceptions and preferences with biologics may facilitate communication between patients and physicians to individualise treatment and improve the experience of people with severe asthma on these treatments.^50^ It is clear that people with severe asthma have important practical and emotional support needs that are currently not being met, but they can be facilitated with a simple change to how information is provided.^21^

Global opinions on asthma control are not always aligned with guidelines.^51^^,^^52^ Gaining insights into views on asthma status would help to improve management and control so that individuals are treated optimally and in line with guidelines.^51^^,^^53^ The Asthma Insight and Management Survey, conducted in the Asia-Pacific region, Canada, Europe, Latin America, and the United States (from 2009 to 2011, N = 10 302), aimed to identify whether the perceptions of people with asthma and the realities of asthma control were in accordance with GINA guidelines.^52^ Overall, individuals inaccurately felt their asthma was well controlled; their views were not consistent with GINA guidelines. The results emphasised that worldwide, individuals lack asthma treatment knowledge and awareness of guidelines.^52^ Similar findings were also found in a global survey of 1333 individuals with severe asthma in 9 countries.^54^ Tools to assess symptom perception and education need to be developed and consistently included in the routine assessment of individuals with asthma.^54^

In an observational, cross-sectional questionnaire from Egypt, Kuwait, Saudi Arabia, Turkey, and the United Arab Emirates (N = 939), uncontrolled asthma was correlated with a high burden and low QoL.^55^ Uncontrolled asthma and increased asthma burden are a global issue (as has also been reported in areas such as the Gulf region, Russia, Thailand, Trinidad, and the United Kingdom), which emphasises the need for further education for those with asthma and inclusion of their perspectives in national guidelines.^30^^,^^53^^,^^56^, ^57^, ^58^, ^59^ In a focus group of 37 people with asthma in Germany, it was highlighted that there was a fear of certain treatments (for example, concerns with cortisone dependency and reduced long-term effectiveness), which leads to treatment misuse owing to a lack of patient knowledge.^60^ Therefore, education on available therapies would reduce concerns and increase adherence.

There is also a need for increased public awareness on the effects of severe asthma to help to reduce misunderstanding of the burden, eg, in the workplace, in order to improve daily living for the individual with severe asthma.^3^ However, it has been estimated that over half of people with asthma do not have the medical knowledge to understand information about their disease.^43^ This indicates that individuals need to be referred to sources of understandable and validated information, such as that supplied by organisations like NHS Choices in the United Kingdom, and patient.information and accredited websites run by asthma charities.^43^ The European Patients’ Academy on Therapeutic Innovation (EUPATI) has created a document aimed at pharmaceutical companies and similar stakeholders to advise how to increase patient involvement, emphasising that close cooperation of stakeholders is necessary for increased transparency and trust.^44^

### Requirement for improvement in stakeholder communication with patients

Adherence to treatments is higher in individuals who are informed and have knowledge about their treatment and care options,^43^ which is encouraged by patient advocacy groups. A systematic review of 55 studies in a wide range of disease areas, including asthma, demonstrated that a positive patient experience is correlated with improved clinical effectiveness and reduced costs.^61^ This review highlighted the need for strategies such as communication training for health care professionals, and for the patient experience to be held as one of the pillars of quality, an initiative that patient advocacy groups can support.^13^^,^^61^ Poor communication among asthma stakeholders has also been documented in other studies (unpublished observation; data on file).^7^^,^^8^^,^^14^ A qualitative survey conducted in France in 30 individuals with asthma treated in primary care found that there are many reasons for a lack of communication, from an individual's inability to digest complicated language provided by their health care professional to poor coordination among professionals, for example pulmonologists and allergists.^8^ There are directive methods that would aid education by increasing accessibility and enhancing knowledge,^44^ such as plain language summaries to complement research articles, which should be made more widely available. Better understanding of the lifestyle and attitudes of those with severe asthma can only aid treatment adherence and interest.^8^

Severe asthma stakeholders should learn from the progress that has been made in the treatment of immune-mediated inflammatory diseases, for instance, psoriasis and rheumatology, which has in recent years had support from active and vocal patient advocacy groups.^62^ Patient-centric treatment goals are now becoming common in psoriasis clinical trials and are being frequently included in new drug assessments, having been issued through organisations such as the UK National Institute for Health and Care Excellence.^63^ Various factors, including the patient advocacy group voice, have led to increased education and awareness of the appropriate clinical and economic data supporting the use of biologic treatment in psoriasis. Patient advocacy groups have been welcomed to the decision-making table to help individuals with psoriasis who often do not have a voice.^62^ Despite the advancements in patient advocacy involvement in asthma therapies, gaps in information still exist. Patient organisations need to be made aware of and have access to resources to provide support during asthma reimbursement discussions.

**Fig. 1:**
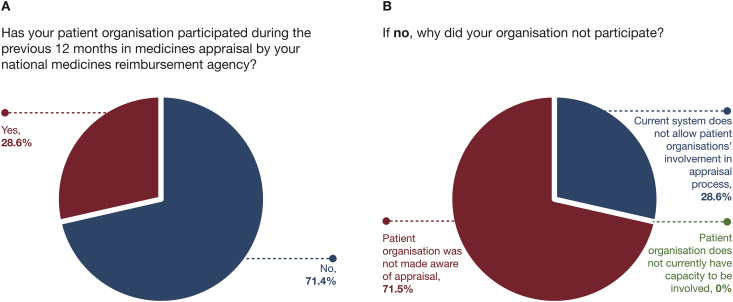
Results from a patient organisation survey conducted across 17 countries.

The Global Allergy and Asthma Patient Platform (GAAPP) survey (previously presented at the American Thoracic Society Congress, 2019) produced data from 19 patient organisations across 17 countries and revealed that few asthma patient organisations (28.6%) participated in medicine appraisals (Fig. 1A). Most patient organisations (71.5%, Fig. 1B) were unaware of the appraisals, but 90.9% would have liked to have been involved (GAAPP Member Reimbursement Policy survey 2019; data on file unpublished observation). It is important that patient advocacy groups and organisations are not only aware of asthma medicine appraisals, but that they also have the resources and capability necessary to participate, in order to not only disseminate individuals’ values but also those of the wider severe asthma community.

Communication between people with severe asthma and other stakeholders is important in preventing avoidable deaths from asthma. The Royal College of Physicians conducted a review in 2013 to evaluate avoidable factors leading to asthma deaths in the United Kingdom.^57^ After evaluation of 3544 available cases of asthma-related deaths in the previous year, asthma was found to be the underlying cause of death in 900 cases, and of these, 195 individuals had died from asthma owing to reasons that were deemed avoidable. If better implementation of asthma guidelines had occurred, 46% of these cases might have been avoided.^57^ Personalised Asthma Action Plans (PAAPs) that improve asthma care had been provided for only 23% of these 195 people, and it was also found that some individuals had not adhered to medical treatment advice, such as not to overuse SABAs (from the prescribing data of 165 individuals, 39% were prescribed more than the recommended maximum of 12 SABA inhalers in the previous year). The recommendations that resulted from these findings included the implementation of a national asthma template to facilitate asthma review, education for carers for managing asthma, and the need for regular assessments to help improve communication at all levels.^57^

While it is important to disseminate guidelines, it is also vital to draw on the knowledge of people with severe asthma to help improve the guidelines and development of treatment options.^16^ Individuals with severe asthma would benefit from an outlet to vocalise their experiences to other stakeholders, particularly during the reimbursement of new medicines. Frequent communication among stakeholders will not only benefit those with severe asthma but also highlight areas where they can improve, including areas such as legislation and policy.^16^ An HTA can evaluate the social and ethical impact of a treatment on an individual, as well as the clinical outcomes and cost-effectiveness; however, patient groups are not often in communication with HTAs (GAAPP Member Reimbursement Policy survey 2019; data on file unpublished observation). Patient groups can help to assist with communication among other stakeholder groups, such as European Patients’ Academy on Therapeutic Innovation (EUPATI) and the European Network for Health Technology Assessment (EUnetHTA). EUPATI has provided a guidance document for patient involvement in HTAs, which involves including patient experts and consulting patient organisations.^16^ The guidance document states that organisations should have proactive communication strategies to obtain a wide range of perspectives from people with asthma.^16^

Collaboration among different asthma specialities, the individuals’ awareness of their own condition and treatment, and increased communication between individuals and stakeholders will help improve outcomes of people with severe asthma and prevent avoidable asthma deaths.^8^

In summary, patients who are well informed and understand what to expect from their asthma care have improved clinical outcomes and higher adherence to treatments than those who are not well informed or do not have a positive experience; improved communication and availability of quality standards ensure that patients are informed about the delivery and quality of their care; and, other channels of communications (and ways to improve communication) should be considered between all stakeholders involved in the delivery of care including patient advocacy groups and regulatory decision makers, and between clinicians (including those involved in drafting guidelines) and patients.

## Conclusion

**Fig. 2:**
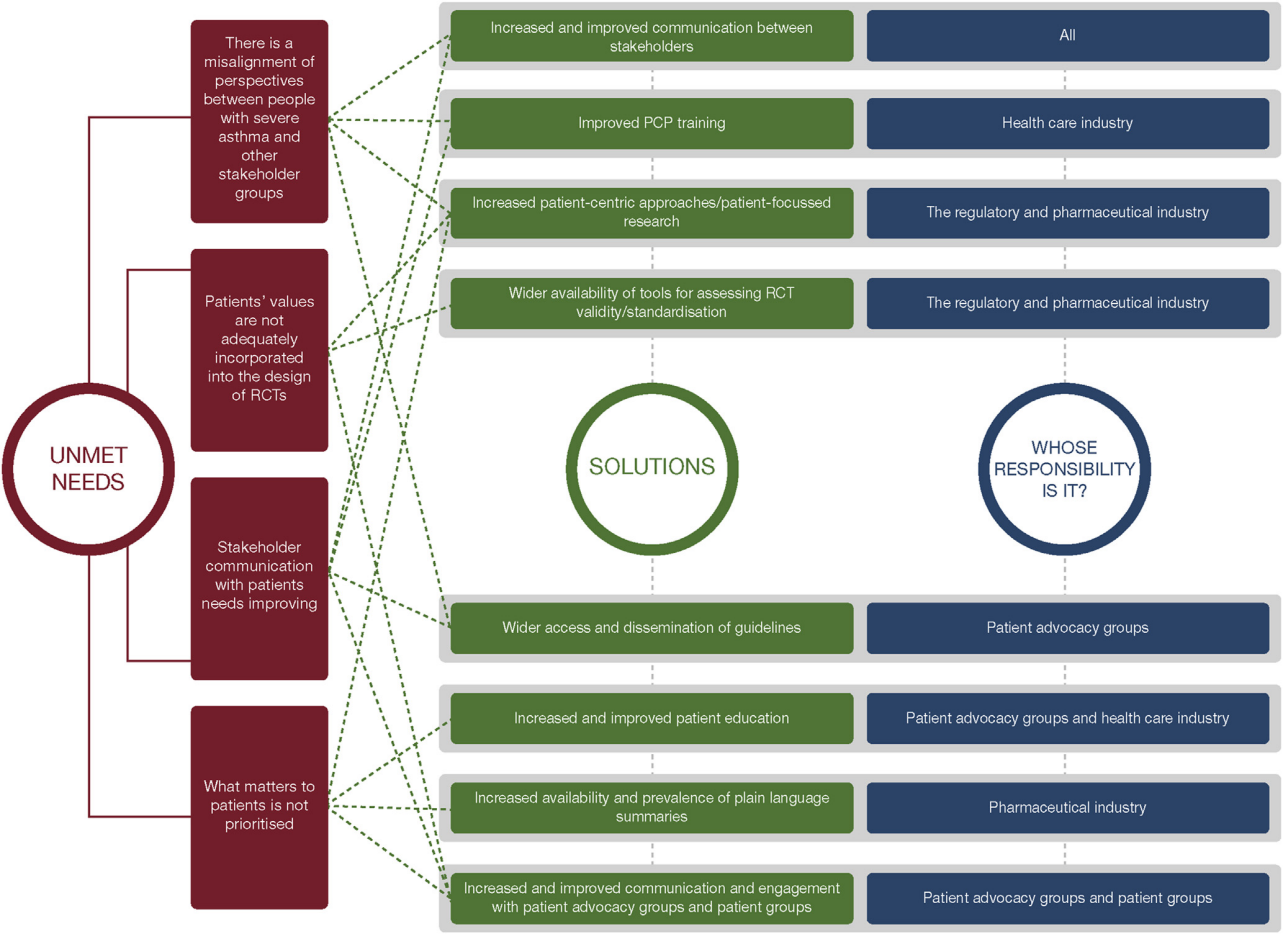
Perspectives on treatment and care from people with severe asthma: current unmet needs and solutions. *PCP: primary care physician; RCT: randomised controlled trial.*

There is a need for all stakeholders in severe asthma care to communicate effectively with each other to improve the lives of people with severe asthma and their caregivers (Fig. 2). Despite the evolution of treatments for asthma and the abundance of national and global guidelines, people with severe asthma are not always being treated according to the best available evidence, nor do they have access to the most appropriate treatments.

## Recommendations

A number of tangible solutions are feasible, including increased patient-focused research, wider availability of tools for assessing RCT validity/standardisation, wider access to and dissemination of guidelines, and increased and improved patient education and health care professional training. All stakeholders at a national level (including patient advocacy groups, HTA organisations and other regulatory and payer bodies, pharmaceutical industry representatives, and clinicians and researchers) need to be mobilised to form a coalition that can incorporate the voice of people with severe asthma into their decision-making and drive change in delivery of patient care, including access to medicines (Fig. 2 and Supplemental Appendix 2).

## Abbreviations

AQLQ, Asthma Quality of Life Questionnaire; EUnetHTA, European Network for Health Technology Assessment; EUPATI, European Patients' Academy on Therapeutic Innovation; FEV_1_, forced expiratory volume in 1 second; GAAPP, Global Allergy and Asthma Patient Platform; GINA, Global Initiative for Asthma; HTA, Health Technology Assessment; OCS, oral corticosteroid(s); OPEN, Observations of Patient Experience in the Nation; PAAP, Personalised Asthma Action Plan; PCP, primary care physician; QoL, quality of life; RCT, randomised controlled trial; SABA, short-acting β_2_-agonist

## Funding

Editorial support was funded by 10.13039/100004325AstraZeneca.

## Availability of data and material

Not applicable.

## Authors’ contributions

All authors contributed equally to the conception, design, acquisition of resources and development of the paper. All authors approved the final version.

## Ethics approval and consent to participate

Not applicable.

## Submission declaration

The authors declare that this is an original work that has not been previously published in any form. The manuscript is not being considered for publication elsewhere.

## Agreement to publish

All authors have read and approved the submitted manuscript and consent to publish the work.

## Declaration of competing interest

MA-A and TW declare that they have no competing interests.

LC is an employee of ASTRAZENECA and owns shares.

JM reports grants, personal fees and non-financial support from ASTRAZENECA and SANOFI; grants from NOVARTIS; personal fees and non-financial support from INMUNOTEK; personal fees from URIACH; grants and non-financial support from GSK, outside the submitted work.
