# Supplementary material for: Perspectives on decisions for treatment and care in severe asthma
Source: World Allergy Organ J. 2021 Jan 16;14(1):100500. doi: 10.1016/j.waojou.2020.100500 (PMC7817505; doi:10.1016/j.waojou.2020.100500)
Supplement: Supplementary file 1 — Multimedia component 1 [file mmc1.pdf]

Supplemental appendix 1: Key findings from included publications

Authors	Year	Title	Journal citation	Country	Publication type/study design	Patient population	Key words	Key points
Alzaabi A, Idrees M, Behbehani N, Khaitov MR, Tunceli K, Urdaneta E, Awad N, Safwat M	2018	Cross-sectional study on asthma insights and management in the Gulf and Russia[a]	*Allergy Asthma Proc.* 2018;39(6): 430-436	Kuwait, Russia, Saudi Arabia, United Arab Emirates	Cross-sectional, multinational, non-interventional, two-phase study	n = 711 patients aged ≥12 years	Asthma; Control; Insights; Perceptions	• Many patients lacked knowledge on asthma control and treatment recommendations<br>• Patient education is needed in the Gulf and Russia
Apps LD, Chantrell S, Majd S, Eglinton E, Singh SJ, Murphy AC, Bradding P, Green RH, Hudson N, Evans RA	2019	Patient perceptions of living with severe asthma: challenges to effective management	*J Allergy Clin Immunol.* Pract 2019; 7(8):2613-2621	UK	Semi structured interviews; qualitative research	n = 29	Difficult asthma; Disease management; Interview; Qualitative; Severe asthma	• Health care professionals need to consider and discuss with patients their perceptions of severe asthma and the relevant treatments faced, especially regarding hospitalisation and education of disease control<br>• There is a need for greater public awareness and education about severe asthma to minimise patient distress, particularly in the work environment
Beharry S, Gidla D, Maharaj A, Bisram S, Sakhamuri S, Seemungal TA, Pinto Pereira LM	2015	Reality and understanding of asthma control[a]	*Chron Respir Dis.* 2015; 12(4):340-346	Trinidad	Cross-sectional	n = 329 adults	Asthma burden; Patient perception; Peak expiratory flow; Revised guidelines; Uncontrolled asthma	• The frequency of patients with actual uncontrolled disease was double what was perceived<br>• Uncontrolled asthma is associated with increased morbidities, work-related difficulties and health care burden
Boonsawat W, Thompson PJ, Zaeoui U, Samosorn C, Acar G, Faruqi R, Poonnoi P	2015	Survey of asthma management in Thailand - the asthma insight and management study[a]	*Asian Pac J Allergy Immunol.* 2015;33(1): 14-20	Thailand	Survey; qualitative research	n = 400 patients aged ≥12 years	Asthma control; Asthma exacerbation; Asthma symptoms; Attitude; Patient burden	• Asthma had a profound impact on patients' wellbeing, despite the availability of effective treatments and evidence-based management guidelines<br>• A large proportion of asthma patients overestimate their asthma control and have inappropriate concepts about asthma treatment
Brusselle G, Michils A, Louis R, Dupont L, Van de Maele B, Delobbe A, Pilette C, Lee CS, Gurdain S, Vancayzeele S, Lecomte P, Hermans C, MacDonald K, Song M, Abraham I	2009	"Real-life" effectiveness of omalizumab in patients with severe persistent allergic asthma: the PERSIST study	*Respir Med.* 2009;103(11):1633-1642	Belgium	Prospective, open-label, observational, multicentre study	n = 158 patients with severe persistent allergic asthma	Allergic asthma; Asthma; Omalizumab; Severe asthma	• The PERSIST study shows better physician-rated effectiveness, greater improvements in QoL, greater reductions in exacerbation rates and greater reductions in health care utilisation than previously reported in efficacy studies<br>• Under real-life conditions, omalizumab is effective as add-on therapy in the treatment of patients with persistent severe allergic asthma

Calderón J, Cherrez A, Ramón GD, Lopez Jove O, Baptist A, Matos E, Morfín Maciel B, Calero E, Sanchez-Borges M, Cherrez S, Simancas-Racines D, Cherrez Ojeda I	2017	Information and communication technology use in asthmatic patients: a cross-sectional study in Latin America[a]	*ERJ Open Res.* 2017;3 (3): 00005-2017	Argentina, Ecuador, Mexico, Peru, Venezuela	Anonymous cross-sectional survey; qualitative research	n = 673	Asthma; Asthma care; Asthma communication; ICT	• ICTs are generally an attractive platform for managing care, communication and interventions to improve asthma care<br>• SMS and e-mail were found to be the preferred ICT forms among users<br>• Social media forms such as WhatsApp and Facebook may also be appropriate for certain types of patient
Canonica GW, Colombo GL, Bruno GM, Di Matteo S, Martinotti C, Blasi F, Bucca C, Crimi N, Paggiaro P, Pelaia G, Passalaqua G, Senna G, Heffler E; SANI Network	2019	Shadow cost of oral corticosteroids-related adverse events: a pharmacoeconomic evaluation applied to real-life data from the Severe Asthma Network in Italy (SANI) registry	*World Allergy Organ J.* 2019;12(1): 100007	Italy	Health economics and outcomes research	SANI database; n = 3,999,600 asthma, n = 199,980 severe asthma	Adverse events; Bone fracture; Chronic kidney disease; Costs; Diabetes; Glaucoma; Obesity; Oral corticosteroids; Pharmacoecono my; Severe asthma	• Co-morbid costs were greater in patients with severe asthma subjected to treatment with high-medium OCS, compared with the moderate and non-asthmatic cohort
Cappuccio A, Napolitano S, Menzella F, Pellegrini G, Policreti A, Pelaia G, Porpiglia PA, Marini MG; SOUND GROUP	2019	Use of narrative medicine to identify key factors for effective doctor-patient relationships in severe asthma[a]	*Multidiscip Respir Med.* 2019;14:26	Italy	Qualitative research	n = 66 physicians writing narratives on 314 patients	Medical education and training; Narrative medicine; Qualitative research; Severe asthma	• Using narrative medicine approaches are useful in daily clinical practice, with the goal of improving the QoL of patients with severe asthma, even in cases in which the doctor-patient relationship is not initially good
Chung KF, Wenzel SE, Brozek JL, Bush A, Castro M, Sterk PJ, Adcock IM, Bateman ED, Bel EH, Bleecker ER, Boulet L-P, Brightling C, Chanez P, Dahlen S-E, Djukanovic R, Frey U, Gaga M, Gibson P, Hamid Q, Jajour NN, Mauad T, Sorkness RL, Teague WG	2014	International ERS/ATS guidelines on definition, evaluation and treatment of severe asthma	*Eur Respir J.* 2014;43(2): 343-373	NA	Guideline revision	NA	Asthma definition; Asthma guidelines; ERS/ATS guidelines; Severe asthma management	• Severe asthma is defined as asthma that requires treatment with high-dose ICS plus a second controller and/or systemic corticosteroids to prevent it from becoming "uncontrolled" or that remains "uncontrolled" despite this therapy<br>• Severe asthma is a heterogeneous condition consisting of phenotypes such as eosinophilic asthma. This guideline provides specific recommendations on the use of sputum eosinophil count and exhaled nitric oxide to guide therapy, as well as treatment with anti-IgE antibody, methotrexate, macrolide antibiotics, antifungal agents and bronchial thermoplasty<br>• Coordinated research efforts for improved phenotyping will provide safe and effective biomarker-driven approaches to severe asthma therapy

Chung LP, Upham JW, Bardin PG, Hew M	2020	Rational oral corticosteroid use in adult severe asthma: a narrative review	*Respirology.* 2020;25(2): 161-172	Australia	Review	NA	Asthma; Biological products; Glucocorticoids; Health promotion; Morbidity	• Given the recent progress in adult severe asthma and new treatment options, judicious appraisal of steroid use is merited<br>• A number of strategies and add-on therapies are now available to treat severe asthma, such as improving guideline adherence, increasing specialist referral, as well as adopting new biologic agents
Chupp GL, Bradford ES, Albers FC, Bratton DJ, Wang-Jairaj J, Nelsen LM, Trevor JL, Magnan A, Ten Brinke A	2017	Efficacy of mepolizumab add-on therapy on health-related quality of life and markers of asthma control in severe eosinophilic asthma (MUSCA): a randomised, double-blind, placebo-controlled, parallel-group, multicentre, phase 3b trial	*Lancet Respir Med.* 2017;5(5): 390-400	Argentina, Belgium, Bulgaria, Canada, Czech Republic, Estonia, France, Germany, Greece, Italy, Netherlands, Norway, Peru, Russia, Slovakia, Spain, Ukraine, UK, USA	Randomised, double-blind, placebo-controlled, parallel-group, multicentre, phase 3b trial	Patients aged ≥12 years with severe eosino-philic asthma; n = 277 placebo, n = 274 mepo-lizumab 100 mg	Add-on therapy; Antibody therapy; IL-5; Mepolizumab; Phase 3b; Quality of life; Safety profile; Severe eosinophilic asthma	• Mepolizumab was associated with significant improvements in HRQoL in patients with severe eosinophilic asthma and had a safety profile similar to that of placebo<br>• Mepolizumab is a favourable add-on treatment option to standard of care in patients with severe eosinophilic asthma
Doyle C, Lennox L, Bell D	2013	A systematic review of evidence on the links between patient experience and clinical safety and effectiveness	*BMJ Open.* 2013;3(1): e001570	UK	Systematic review	A wide range of demo-graphics and age groups from 55 studies	Asthma; Patient care; Patient experience; Patient outcomes; Patient safety; Systematic review	• Patient experience is consistently positively associated with patient safety and clinical effectiveness across a wide range of disease areas, study designs, settings, population groups and outcome measures<br>• The data presented support the case for the inclusion of patient experience as one of the central pillars of quality in health care

Farne HA, Wilson A, Powell C, Bax L, Milan SJ	2017	Anti-IL5 therapies for asthma	*Cochrane Database Syst Rev.* 2017;9(9): CD010834	International	Systematic review	n = 6000 adults and children with asthma (from 13 eligible studies)	Asthma; Benralizumab; Mepolizumab; RCT; Reslizumab; Systematic review	• The use of anti-IL-5 treatments as an adjunct to standard of care in people with severe eosinophilic asthma and poor control is supported by the review<br>• These biologics roughly halve the rate of asthma exacerbations in this population<br>• There is limited evidence for improved HRQoL scores and lung function, which may not meet clinically detectable levels<br>• There were no safety concerns regarding mepolizumab or reslizumab, and no excess serious AEs with benralizumab, although there remains a question over AEs significant enough to prompt discontinuation<br>• Further research is needed on biomarkers for assessing treatment response, optimal duration and long-term effects of treatment, risk of relapse on withdrawal, non-eosinophilic patients, children (particularly under 12 years), and comparing anti-IL-5 treatments to each other and, in people eligible for both, to anti-IgE. For benralizumab, future studies should closely monitor rates of AEs prompting discontinuation
Foster JM, McDonald VM, Guo M, Reddel HK	2017	"I have lost in every facet of my life": the hidden burden of severe asthma	*Eur Respir J.* 2017;50(3): 1700765	Australia	Semistructured interviews; qualitative research	n = 25 adults with severe asthma	Asthma burden; Emotional distress; Patient experience; Patient support; Severe asthma; Treatment burden	• Severe asthma imposes long-term, debilitating burdens and should be considered differently to milder disease. There is an urgent need to improve practical and emotional support services for patients and their carers
George M, Keddem S, Barg FK, Green S, Glanz K	2015	Urban adults' perceptions of factors influencing asthma control[a]	*J Asthma.* 2015;52(1): 98-104	USA	Semistructured interviews; qualitative research	n = 35 adults with persistent asthma (94% black; 71% female; 71% with uncontrolled asthma)	Community; Environmental; Minority; Qualitative; Triggers	• Compared with participants with controlled asthma, uncontrolled participants reported overusing short-acting β-agonists, underusing ICS, rejecting medical and trigger remediation advice, having more negative experiences with primary care providers, and preferring more unconventional strategies to prevent or manage asthma symptoms<br>• Personal health beliefs about control can undermine adherence to medical advice; new patient-centric models of care may be required to modify these beliefs

Hannane A, Misane L, Devouassoux G, Colin C, Letrilliart L	2019	Asthma patients' perception on their care pathway: a qualitative study	*NPJ Prim Care Respir.* Med 2019; 29(1):9	France	Interviews; qualitative research	n = 30	Care; Disease management; Education; Patient communication; Qualitative; Quality of life	• Asthma management at diagnosis and follow-up phases proved to be unstructured and were associated with poor patient education<br>• Poor management was due to: patients' ambivalence (in relation to illness and treatments), poor communication between patients and health care professionals (lack of listening and use of inappropriate vocabulary by physicians, under-reporting of alternative medicine use by patients) and weak cooperation between professionals (limited to interaction between the GP and the specialist, either pulmonologist or allergist)<br>• Asthma management would probably benefit from a more coordinated care pathway at each phase of the disease that is consistent with the expectations and goals of the patients
Hossny E, Caraballo L, Casale T, El-Gamal Y, Rosenwasser L	2017	Severe asthma and quality of life	*World Allergy Organ J.* 2017;10(1): 28	NA	Review	NA	Adherence; Biologics; Monoclonal antibodies; Quality of life; Severe asthma	• The most important clinical parameter affecting the QoL of patients with bronchial asthma is disease severity<br>• A number of biologics have been developed to treat asthma characterised by eosinophilic inflammation with or without antigen-specific IgE<br>• An anti-IgE and two anti-IL-5 monoclonal antibodies are approved for the treatment of severe asthma and were correlated with better QoL in several trials<br>• QoL in severe asthma could also be improved by achieving better adherence to therapy, potentiating health education, addressing risk factors, and targeting social and psychological domains
Katsaounou P, Odemyr M, Spranger O, Hyland ME, Kroegel C, Conde LG, Gore R, Menzella F, Domingo Ribas C, Morais-Almeida M, Gasser M, Kasujee I	2018	Still Fighting for Breath: a patient survey of the challenges and impact of severe asthma	*ERJ Open Res.* 2018;4(4): 00076-2018	Brazil, Canada, France, Germany, Japan, Italy, Portugal, Spain, UK	Global survey; qualitative research	n = 1333 adult patients (aged >18 years) and caregivers of children (aged 6–17 years) with severe persistent asthma	Burden; Control; Perception; Qualitative; Quality of life	• A large discrepancy was observed between the proportion of patients who perceived their asthma to be well controlled (42%) and the proportion of patients who reported to be well controlled as per the GINA assessment (6%)<br>• Although most patients perceived their asthma to be controlled, many experienced frequent symptoms that affected their daily lives
Kerdel F, Zaiac M	2015	An evolution in switching therapy for psoriasis patients who fail to meet treatment goals	*Dermatol Ther.* 2015; 28(6):390-403	NA	Review	NA	Disease management; Efficacy; Psoriasis; Strategies; Switching; Treatment goals	• Failure on one agent does not predict future treatment failure with different agents, and prompt alteration of treatment should be a priority for patients<br>• An essential component to maximising treatment success is communication between patients and practitioners to develop realistic treatment goals

Khadadah M, Mahboub B, Al-Busaidi NH, Sliman N, Soriano JB, Bahous J	2009	Asthma insights and reality in the Gulf and the near East	*Int J Tuberc Lung Dis.* 2009;13(8): 1015-1022	Jordan, Kuwait, Lebanon, Oman, United Arab Emirates	Survey; qualitative research	n = 1000 asthma patients	Asthma; Burden; Gulf and Near East; Undertreatment	• Current levels of asthma control in the Gulf and near East fall far short of the goals specified in guidelines for asthma management; work/school absence and health service use was high while peak expiratory flow and lung function use was low
Larenas-Linnemann D, Fernández-Vega M, Rodríguez-González M, Cano-Salas MC, Luna-Pech JA, Ortega-Martell JA, Del Rio-Navarro B, López-Estrada EC, Romero-Lombard J, Vázquez-García JC, Salas-Pérez J	2019	An online survey detected knowledge gaps and cost-saving opportunities in asthma maintenance treatment among allergists, pulmonologists, ENTs and primary care	*World Allergy Organ J.* 2019;12(12): 100084	Mexico	Survey; qualitative research	247 allergists, 83 pulmon-ologists, 14 ENTs, 135 paediatricians and 37 GPs finished the survey	Allergist; Asthma treatment; Education; Inhaled corticosteroid; Long-acting beta agonist; Omalizumab; Paediatrician; Pulmonologist; Spirometry; Theophylline; Tiotropium bromide	• Surveyed physicians tended to over-treat milder asthma cases<br>• Caution should be taken in the promotion of the SMART (single maintenance-and-reliever-treatment) approach, which can only be done with ICS-formoterol. Many physicians opt for other combinations that are not suitable<br>• Among all surveyed specialities there is ample room for improvement in mild and severe asthma management
Lingner H, Burger B, Kardos P, Criée CP, Worth H, Hummers-Pradier E	2017	What patients really think about asthma guidelines: barriers to guideline implementation from the patients' perspective	*BMC Pulm Med.* 2017; 17(1):13	Germany	Qualitative exploratory design, topic-centred focus group discussions	n = 13 men, n = 24 women aged 20–77 with asthma	Asthma; Barriers; Burden; Communication; Disease management; Guidelines; Patient care; Treatments	• Patients had concerns about: rejection of therapy components; lack of time or money for optimal treatment; insufficient involvement in therapy choices; suboptimal communication between health care professionals; difficulties with recommendations conflicting with daily life<br>• Patients wanted more time with doctors to discuss difficulties and all aspects of living with an impairing condition<br>• In order to advance guideline implementation and improve asthma treatment, the patients’ perspective needs to be considered before drafting new versions
Lisspers K, Teixeira P, Blom C, Kocks J, Ställberg B, Price D, Chavannes N	2016	Are pharmacological randomised controlled clinical trials relevant to real-life asthma populations? A protocol for an UNLOCK study from the IPCRG	*NPJ Prim Care Respir Med.* 2016; 26:16016	International	Protocol; observational study to come	Patients in primary care databases	Guidelines; Population; Randomised controlled trial; Real world; Trial representative-ness	• Previous findings suggest that the level of representation of the real-world population with asthma and COPD in RCTs may be lower than desirable<br>• This study can help inform the development of future clinical practice guidelines but also the way asthma clinical trials are being designed and implemented<br>• The study will tell us more about the patients who are not eligible for these major RCTs, and it may also provide new insights into why the control of asthma in many patients does not appear to improve in spite of the implementation of treatment strategies recommended in the guidelines

Liu D, Ahmet A, Ward L, Krishnamoorthy P, Mandelcorn ED, Leigh R, Brown JP, Cohen A, Kim H	2013	A practical guide to the monitoring and management of the complications of systemic corticosteroid therapy	*Allergy Asthma Clin Immunol.* 2013;9(1):30	NA	Review	NA	Adrenal suppression; Adverse events; Corticosteroids; Cushing's syndrome; Glaucoma; Glucocorticoid-induced osteoporosis; Glucocorticoids; Hyperglycaemia; Side effects; Systemic	• Patients should be informed about the AEs associated with systemic corticosteroid use and should be advised on lifestyle modification strategies that may help reduce the risk of these events<br>• Patients should also be instructed to seek medical attention if they experience signs and symptoms of steroid-related AEs and should be advised to carry a steroid treatment card that can be shown to all health care professionals involved in their care and management
Lötvall J, Akdis CA, Bacharier LB, Bjermer L, Casale TB, Custovic A, Lemanske RF Jr, Wardlaw AJ, Wenzel SE, Greenberger PA	2011	Asthma endotypes: a new approach to classification of disease entities within the asthma syndrome	*J Allergy Clin Immunol.* 2011;127(2): 355-360	International	Consensus report	NA	Asthma; Cluster analysis; Endotype; Epidemiology; Pathophysiology; Phenotype	• Patients with different characteristics/endotypes may respond differently even to currently available treatments<br>• A major unmet need in asthma lies with delivering mechanism-specific treatments that are highly effective in specific endotypes of asthma; trials should be designed with specific endotypes in mind
Maspero JF, Jardim JR, Aranda A, Tassinari CP, Gonzalez-Diaz SN, Sansores RH, Moreno-Cantu JJ, Fish JE	2013	Insights, attitudes, and perceptions about asthma and its treatment: findings from a multinational survey of patients from Latin America	*World Allergy Organ J.* 2013;6(1):19	Argentina, Brazil, Mexico, Puerto Rico, Venezuela	Face-to-face interviews; qualitative research	n = 2169 adults or parents of children with asthma	Asthma; Controlled; Exacerbation; Guidelines; Patient burden; Symptoms	• There is a discrepancy between patient perception of asthma control and guideline-mandated criteria; asthma patients in Latin America overestimated their degree of asthma control<br>• Additional education is required to teach patients that, by more closely following asthma management strategies outlined by current guidelines, more patients can achieve adequate asthma control
Menzies-Gow A, Canonica G-W, Winders TA, Correia de Sousa J, Upham JW, Fink-Wagner A-H	2018	A charter to improve patient care in severe asthma	*Adv Ther.* 2018;35(10): 1485-1496	International	Commentary	NA	Health care policy; Patient advocacy; Patient care; Respiratory; Severe asthma	• A patient charter to improve care in severe asthma has been drawn up to ensure: timely and straightforward referral, timely and formal diagnosis by a multidisciplinary team, consistent and quality support and care, and a reduction in the reliance on OCS
Mercieca-Bebber R, King MT, Calvert MJ, Stockler MR, Friedlander M	2018	The importance of patient-reported outcomes in clinical trials and strategies for future optimization	*Patient Relat Outcome Meas.* 2018; 9:353-367	Australia	Review	NA	Clinical trials as topic; Patient-reported outcomes; Quality of life; Research practices; Trial conduct	• Researchers and clinical trial investigators must implement evidence-based strategies to promote high-quality PRO data collection, analysis, and reporting of PRO evidence<br>• PRO aspects of trial protocols should be developed in accordance with the SPIRIT-PRO guidance<br>• PRO findings should be published according to CONSORT-PRO and ISOQOL PRO reporting guidelines in a timely manner

Mungan D, Aydin O, Mahboub B, Albader M, Tarraf H, Doble A, Lahlou A, Tariq L, Aziz F, El Hasnaoui A	2018	Burden of disease associated with asthma among the adult general population of five Middle Eastern countries: results of the SNAPSHOT program[a]	*Respir Med.* 2018;139: 55-64	Egypt, Turkey and a Gulf cluster (Kuwait, Saudi Arabia, United Arab Emirates)	Observational, cross-sectional	n = 939	Asthma; Burden of disease; Middle East; SNAPSHOT	• Uncontrolled asthma imposes a significant burden in these Middle Eastern countries resulting in increased frequency of health care use, lower QoL and a higher impact on daily life compared with controlled asthma
Nathan RA, Thompson PJ, Price D, Fabbri LM, Salvi S, González-Díaz S, Maspero JF, Moreno-Cantu JJ, Fish JE, Murphy K	2015	Taking aim at asthma around the world: global results of the asthma insight and management survey in the Asia-Pacific Region, Latin America, Europe, Canada, and the United States	*J Allergy Clin Immunol.* Pract 2015; 3(5):734-742	20 countries in North America, Europe, Latin America and Asia-Pacific region	Global survey; qualitative research	n = 10302	Asthma; Control; Guidelines; Management; Survey; Symptoms; Treatment	• Patients exhibited a lack of knowledge and conviction for treatment recommendations and guidelines that was relatively uniform across the regions, similar to earlier survey findings
Nielsen CP, Lauritsen SW, Kristensen FB, Bistrup ML, Cecchetti A, Turk E; European network for Health Technology Assessment Work Package 6 Partners	2009	Involving stakeholders and developing a policy for stakeholder involvement in the European network for health technology assessment, EUnetHTA	*Int J Technol Assess Health Care.* 2009;25 (Suppl. 2): 84-91	Europe	Commentary, basic research	NA	Decision making; Health technology assessment; Interest groups; Stakeholder	• Stakeholder involvement in EUnetHTA is necessary to ensure the legitimacy and prospects for utilisation of EUnetHTA and its products<br>• The EUnetHTA stakeholder meeting can be considered a successful experience of dialogue between EUnetHTA and stakeholders<br>• Continued attention should be given to achieving balanced stakeholder representation
Norman G, Faria R, Paton F, Llewellyn A, Fox D, Palmer S, Clifton I, Paton J, Woolacott N, McKenna C	2013	Omalizumab for the treatment of severe persistent allergic asthma: a systematic review and economic evaluation	*Health Technol Assess.* 2013; 17(52):1-342	UK	Systematic review	NA	Asthma; Economic evaluation; Omalizumab; Review; Treatment; UK	• Omalizumab reduces the incidence of clinically significant exacerbations in adults/children, with benefits on other outcomes in adults<br>• Limited, underpowered subgroup evidence exists that omalizumab reduces exacerbations and OCS requirements in adults on OCSs. Evidence in children is weaker and more uncertain<br>• The incremental cost-effectiveness ratios are above conventional National Health Service thresholds of cost-effectiveness. The key drivers of cost-effectiveness are asthma-related mortality risk and, to a lesser extent, HRQoL improvement and OCS-related AEs<br>• An adequately powered double-blind RCT in both adults and children on maintenance OCSs and an individual patient data meta-analysis of existing trials should be considered. A registry of all patients on omalizumab should be established

Nunes C, Pereira AM, Morais-Almeida M	2017	Asthma costs and social impact	*Asthma Res Pract.* 2017; 3:1	International	Review	NA	Asthma; Burden; Control; Costs; Morbidity; Mortality; Socio-economic	• Despite the introduction of guidelines, the estimated costs of asthma can still be considered substantial, stressing the need for more comprehensive approaches that can be fully implemented in different settings<br>• Although it is recognised that asthma is a costly illness, the total cost of asthma to society has not been estimated in most countries
Patel MR, Wheeler JR	2014	Physician-patient communication on cost and affordability in asthma care. Who wants to talk about it and who is actually doing it[a]	*Ann Am Thorac Soc.* 2014;11(10): 1538-1544	USA	Secondary data analysis	n = 422 African-American women with persistent asthma	Cost-related nonadherence; Financial burden; Patient preferences; Self-management	• An imbalance is evident between patients who would like to discuss cost with their doctor and those who actually do<br>• Patients are interested in low-cost options and a venue for addressing their concerns with a care provider
Peláez S, Bacon SL, Lacoste G, Lavoie KL	2016	How can adherence to asthma medication be enhanced? Triangulation of key asthma stakeholders' perspectives	*J Asthma.* 2016;53(10): 1076-1084	Canada	Qualitative, multiple case study	n = 38 asthma stakeholders (n = 13 patients, n = 13 pulmonologist physicians, n = 12 allied health care professionals)	Education; Facilitators; Focus groups; Health care professionals; Intervention; Patients; Physicians	• Asthma medication adherence is a complex process and successful interventions aimed at its improvement would benefit from: (a) making an effort to understand patients' experiences and negotiate the treatment regimen rather than imposing recommendations; (b) considering treatment as a shared responsibility involving the patient, the health care professional(s) and the patients' social networks; and (c) taking into account different stakeholders' concerns, needs, perspectives and knowledge
Price D, Bjermer L, Bergin DA, Martinez R	2017	Asthma referrals: a key component of asthma management that needs to be addressed	*J Asthma.* Allergy 2017;10: 209-223	International	Review	NA	Asthma; Disease management; Primary care physicians; Referral; Specialisation	• To achieve appropriate referrals, there needs to be additional proper dissemination, translation, training, implementation and adherence of appropriate asthma guidelines that suit the health care landscape<br>• An effective specialist referral system requires better coordination between health care providers and patients in different settings
Roche N, Campbell JD, Krishnan JA, Brusselle G, Chisholm A, Bjermer L, Thomas M, van Ganse E, van den Berge M, Christoff G, Quint J, Papadopoulos NG, Price D	2019	Quality standards in respiratory real-life effectiveness research: the REal Life EVidence AssessmeNt Tool (RELEVANT): report from the Respiratory Effectiveness Group-European Academy of Allergy and Clinical Immunology Task Force	*Clin Transl.* Allergy 2019;9:20	International	Tool validation; report	NA	Asthma; Comparative effectiveness; Database; Observational studies; Quality standards	• Although highlighting a general lack of high-quality real-life effectiveness observational research on these clinically important topics, the analysis provided insights into how identified observational studies might inform asthma guidelines developers and clinicians<br>• Overall, the REal Life EVidence AssessmeNt Tool appeared reliable and easy to use by expert reviewers

Sturdy PM, Victor CR, Anderson HR, Bland JM, Butland BK, Harrison BD, Peckitt C, Taylor JC; Mortality and Severe Morbidity Working Group of the National Asthma Task Force	2002	Psychological, social and health behaviour risk factors for deaths certified as asthma: a national case-control study	*Thorax.* 2002;57(12): 1034-1039	UK	Community-based, case-control study	n = 533 cases comprising 78% of all asthma deaths under age 65 years; n = 533 age-matched controls	Asthma; Behaviour; Case-control; Death; Disease burden; Psychosocial factors; Risk factors	• There was an apparently high burden of psychosocial problems in both cases and controls<br>• The associations between health behaviour, psychosocial factors and asthma death are varied and complex, with a limited number of factors showing positive relationships
Sullivan PW, Smith KL, Ghushchyan VH, Globe DR, Lin SL, Globe G	2013	Asthma in USA: its impact on health-related quality of life[a]	*J Asthma.* 2013;50(8): 891-899	USA	Survey; qualitative research	n = 46534 individuals without asthma, with asthma currently or previously had asthma	Asthma control; EQ-5D; Health function; Health related quality of life; Health status; National burden; SF-12	• Asthma itself, and especially indicators of poor asthma control, were associated with a deleterious effect on health function, preference-based HRQoL and self-perceived health status
Tapp H, Derkowski D, Calvert M, Welch M, Spencer S	2017	Patient perspectives on engagement in shared decision-making for asthma care	*Fam Pract.* 2017;34(3): 353-357	USA	Case study	n = 16 patients	Dissemination; Focus groups; Patient advisory boards; Patient engagement; Patient experience; Primary care	• Patient engagement directly influenced multiple aspects of the study, including study design, implementation, data analysis and dissemination through incorporation of the patients' and caregivers' input and concerns
Thompson PJ, Salvi S, Lin J, Cho YJ, Eng P, Abdul Manap R, Boonsawat W, Hsu J-Y, Faruqi RA, Moreno-Cantu JJ, Fish JE, Ho JC-M	2013	Insights, attitudes and perceptions about asthma and its treatment: findings from a multinational survey of patients from 8 Asia-Pacific countries and Hong Kong[a]	*Respirology.* 2013;18(6): 957-967	Australia, China, Hong Kong, India, Malaysia, Singapore, South Korea, Taiwan, Thailand	Survey; qualitative research	n = 3630 asthma patients (≥12 years)	Controlled; Exacerbation; Patient burden; Symptoms	• Asthma has a profound impact on patients' wellbeing despite the availability of effective treatments and evidence-based management guidelines<br>• Substantial differences across the surveyed countries exist, suggesting unmet, country-specific cultural and educational needs<br>• A large proportion of asthma patients overestimate their level of control
Travers J, Marsh S, Williams M, Weatherall M, Caldwell B, Shirtcliffe P, Aldington S, Beasley R	2007	External validity of randomised controlled trials in asthma: to whom do the results of the trials apply?	*Thorax.* 2007;62(3): 219-223	New Zealand	Survey; qualitative research	n = 749 (n = 179 with asthma) completed survey	Clinical trial; Eligibility criteria; Patient population; Real world; Survey	• The major asthma RCTs on which the GINA guidelines are based may have limited external validity as they have been performed on highly selected patient populations<br>• Most of the participants with current asthma on treatment in the community would not have been eligible for these RCTs

Vamos M, Kolbe J	1999	Psychological factors in severe chronic asthma	*Aust N Z J Psychiatry.* 1999;33(4): 538-544	Australia, New Zealand	Questionnaires and structured interviews	n = 80 patients with severe asthma aged 14–76 years	Anxiety; Asthma; Depression; Life-threatening asthma; Psychosocial factors	• Levels of asthma knowledge were dangerously low, despite apparently adequate educational initiatives<br>• Patients with severe asthma have high levels of distress, particularly of anxiety, even between attacks<br>• Patients' attitudes to their illness are multifactorial and are significantly correlated with emotional distress, morbidity indices and some demographic factors
Warner K, See W, Haerry D, Klingmann I, Hunter A, May M	2018	EUPATI guidance for patient involvement in medicines research and development (R&D); guidance for pharmaceutical industry-led medicines R&D	*Front Med (Lausanne).* 2018;5:270	Europe	Guidance document	NA	EUPATI; Guidance; Medicines development; Patient engagement; Patient involvement; Pharmaceutical industry-led medicines R&D	• The pharmaceutical industry should strive to involve patients early in medicine development, preferably before the clinical development phases<br>• Pharmaceutical companies need to continue to evolve their processes and governance infrastructure to integrate patient involvement, and patient organisations need to provide relevant input and increase their ability to identify individual patients who are interested in getting involved
Zimmerman GM, Savage LM, Chandler DC, Maccarone Buonfigli M	2005	Psoriatic arthritis and psoriasis: role of patient advocacy organisations in the twenty first century	*Ann Rheum Dis*. 2005;64 (Suppl. 2): ii93-ii100	International	Report	NA	Education; Health politics; Lobbying; Patient advocacy group; Public awareness	• Patient advocacy organisations play a vital role in the treatment of psoriasis and psoriatic arthritis; they provide support for patients and physicians, and lobby for better treatments<br>• Whatever their size, as their roles have come to be recognised in the health care community, the patient advocacy organisations welcome being invited to the decision-making table

[a]Denotes the articles were sourced through a literature search.
AE: adverse event; ATS: American Thoracic Society; COPD: chronic obstructive pulmonary disease; ENT: ear, nose and throat specialist; EQ-5D: European quality-of-life 5-dimensional instrument; ERS: European Respiratory Society; EUnetHTA: European Network for Health Technology Assessment; *EUPATI: European Patients' Academy on Therapeutic Innovation; GINA: Global initiative for asthma; GP: general practitioner; HRQoL: health-related quality of life; ICS: inhaled corticosteroid(s); ICT: information and communication technology; IgE: immunoglobulin E; IL-5: interleukin 5; IPCRG: International Primary Care Respiratory Group; ISOQOL: International Society for Quality of Life Research; NA: not applicable; OCS: oral corticosteroid(s); PRO: patient-reported outcome; QoL: quality of life; RCT: randomised controlled trial; SF-12: short-form health survey 12; SMS: short message service; UNLOCK: Uncovering and Noting Long-Term Outcomes in COPD and asthma to enhance knowledge.*
