# Supplementary material for: Perspectives on decisions for treatment and care in severe asthma
Source: World Allergy Organ J. 2021 Jan 16;14(1):100500. doi: 10.1016/j.waojou.2020.100500 (PMC7817505; doi:10.1016/j.waojou.2020.100500)
Supplement: Supplementary file 2 — Multimedia component 2 [file mmc2.pdf]

Supplementary appendix 2: People with severe asthma's perspectives on treatment and care: current unmet needs and solutions

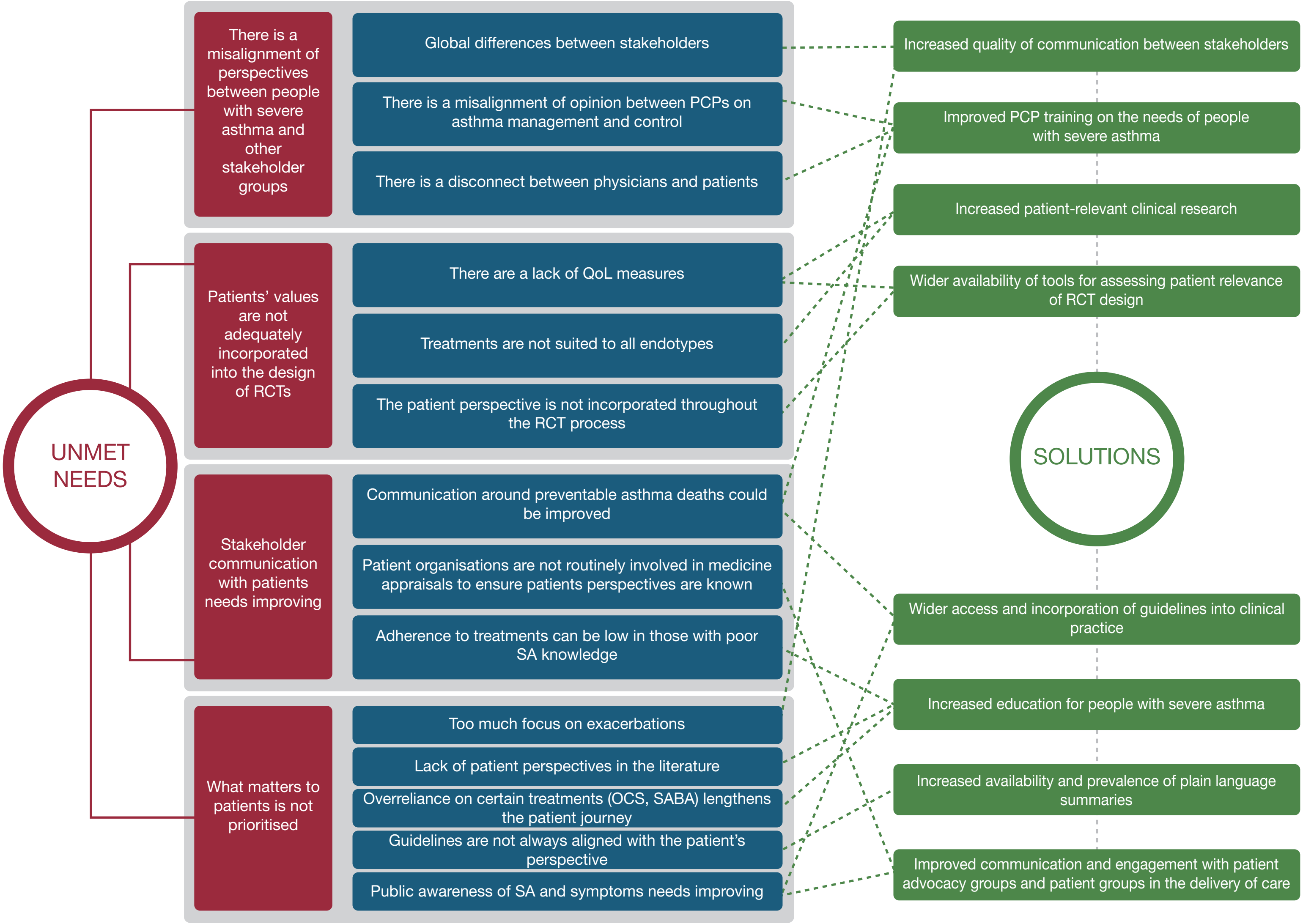

OCS: oral corticosteroid; PCP: Primary care physician; QoL: quality of life; RCT: randomised controlled trial; SA: severe asthma; SABA: short-acting β-agonist.
